# Psychological Well-Being of Older Adults With Cognitive Deterioration During Quarantine: Preliminary Results From the GeroCovid Initiative

**DOI:** 10.3389/fmed.2021.715294

**Published:** 2021-09-22

**Authors:** Alessandra Coin, Maria Devita, Caterina Trevisan, Francesca Biasin, Camilla Terziotti, Susanna Del Signore, Stefano Fumagalli, Pietro Gareri, Alba Malara, Enrico Mossello, Stefano Volpato, Fabio Monzani, Giuseppe Bellelli, Gianluca Zia, Anette Hylen Ranhoff, Raffaele Antonelli Incalzi

**Affiliations:** ^1^Geriatrics Division, Department of Medicine (DIMED), Azienda Ospedale Università di Padova, University of Padova, Padua, Italy; ^2^Department of General Psychology (DPG), University of Padova, Padua, Italy; ^3^Bluecompanion Ltd, London, United Kingdom; ^4^Geriatric Intensive Care Unit, Department of Experimental and Clinical Medicine, University of Firenze, Firenze, Italy; ^5^Center for Cognitive Disorders and Dementia - Catanzaro Lido, ASP Catanzaro, Catanzaro, Italy; ^6^Scientific Committee of National Association of Third Age Residences (ANASTE) Calabria, Lamezia Terme, Italy; ^7^Department of Medical Science, University of Ferrara, Ferrara, Italy; ^8^Geriatrics Unit, Department of Clinical and Experimental Medicine, University of Pisa, Pisa, Italy; ^9^Acute Geriatric Unit, School of Medicine and Surgery, San Gerardo Hospital, University of Milano-Bicocca, Monza, Italy; ^10^Department of Clinical Science, Norway and Diakonhjemmet Hospital, University of Bergen, Oslo, Norway; ^11^Unit of Geriatrics, Department of Medicine, Campus Bio-Medico University and Teaching Hospital, Rome, Italy

**Keywords:** COVID-19, dementia, psychological well-being, older adults, distress

## Abstract

**Objectives:** The spread of COVID-19 has undeniably unsettled the social, psychological and emotional life of the entire world population. Particular attention should be paid to older adults with dementia, given their vulnerability to emotional stressors. The aim of this retrospective study is to evaluate the impact of the first wave quarantine related to Covid-19 on psychological and affective well-being of older adults with mild/major neurocognitive disorders and of their caregivers.

**Methods:** Data on participants' assessment before the quarantine (PREQ) were retrospectively collected. Patients with Mild Cognitive Impairment (MCI) or dementia were recruited from different Centers for Cognitive Decline and Dementia in Italy. During the quarantine, psychological and affective well-being were evaluated by phone through the administrations of scales measuring anxiety and depression (DASS), perceived stress (PSS), coping strategies (COPE) and the caregivers' burden (CBI). The scales' results were compared across participants' PREQ cognitive level (Mini Mental State Examination, MMSE ≥25, 23–24, and ≤ 22) with multiple linear regression models.

**Results:** The sample included 168 patients (64% women) with a mean age of 79 ± 7 years. After adjusting for potential confounders, more severe cognitive impairment was independently associated with higher DASS and PSS score, and poorer coping strategies (*p* < 0.05). Cognitive functioning was also inversely associated with CBI.

**Conclusions:** The impact of the quarantine on the psycho-affective well-being of individuals with MCI and dementia and on caregivers' burden varies according to the PREQ cognitive functioning with more severely impaired patients having worse outcomes.

## Key points

- During the pandemic, individuals cognitively more impaired showed more severe depressive and anxious symptoms, compared to those with better cognitive functioning.
- Individuals with greater cognitive impairment showed worse “positive attitude” and “problem orientation” coping strategies, as compared to those with better cognitive functioning.
- Heavier caregiving burden was observed in caregivers of individuals with more severe cognitive impairment.

## Introduction

The Italian population, as well as the entire world, is in a delicate historical phase as the spread of novel Coronavirus (COVID-19) is requiring important clinical, social and economic interventions in order to limit escalation of the disease and safeguard individuals' health. Quarantine, social distancing and community containment are the public health measures that have been adopted to isolate people and prevent person-to-person transmission of the disease (1). If, on the one hand, these measures are fundamental to reduce the transmission of COVID-19 and its serious consequences on people's health, on the other hand they may have significant negative sequelae. An increasing number of studies are documenting the impact of COVID-19 itself and of the forced social isolation on psychological well-being (2–4). In this context, it has been recently highlighted the importance of drawing attention also to the psychological consequences of the pandemic on older adults. As at the date worldwide acknowledged, scrupulous consideration should be given to older adults, who represent the section of the population with the highest rate of mortality linked to this virus (5). More specifically, attention should be made to the “frailest among the frail,” the individuals with dementia (6). Different data on the psychological and/or behavioral effects of COVID-19 on individuals with dementia have been reported so far (3, 7). Nonetheless, some questions remain unanswered: do people with Mild Cognitive Impairment (MCI) and dementia experience anxiety and depression because of the COVID-19 pandemic? If so, is there an association between the extent of cognitive impairment and psychological well-being? And how may cognitively impaired older individuals may cope with a sudden and unexpected event, such as COVID-19? The aim of this study is to evaluate the impact of spring 2020 lockdown period in Italy due to COVID-19 on psychological and affective well-being of older adults with different levels of cognitive impairment and of their caregivers. We hypothesized that the COVID-19 quarantine may have had a stronger impact on individuals with worse cognitive performance in terms of affective symptoms and coping strategies and on their caregivers in terms of perceived burden.

## Materials and Methods

### Study Design

**Figure 1:**
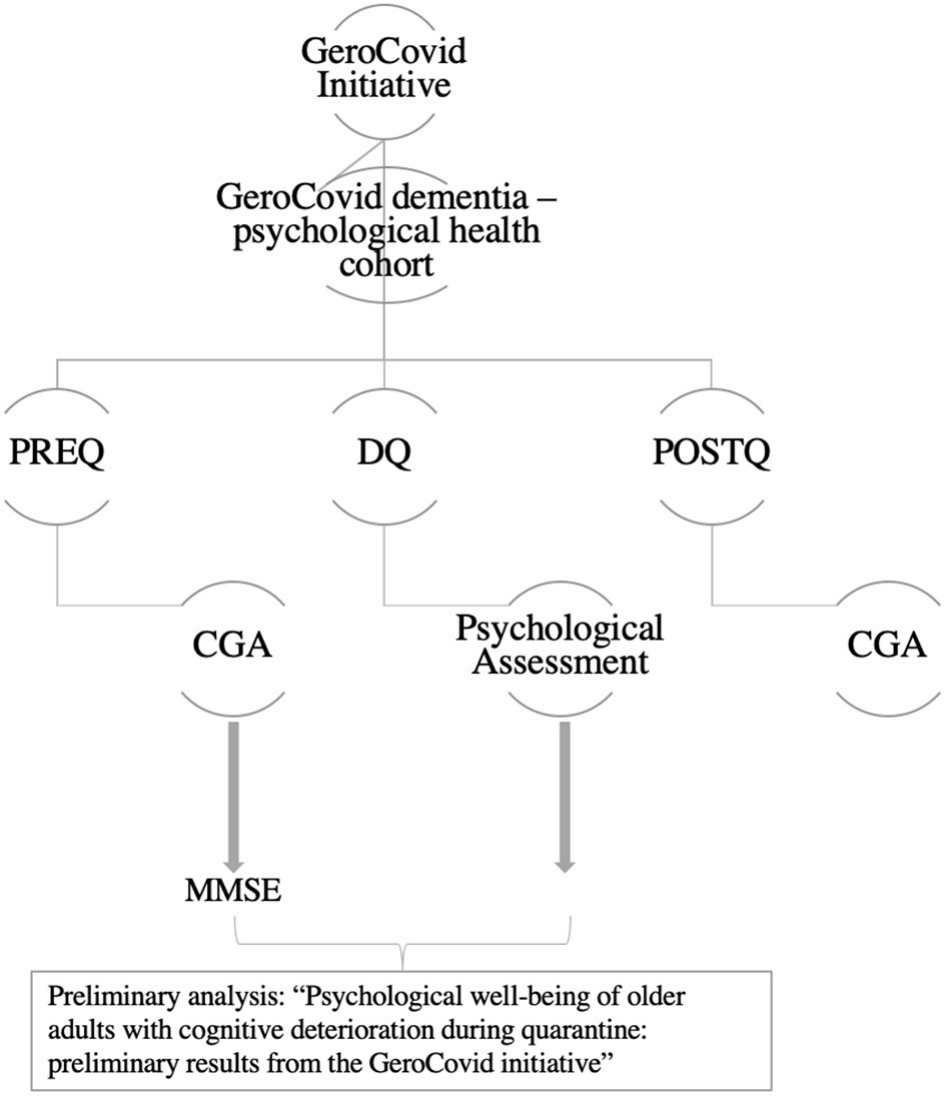
Schematic representation of study design. PREQ, evaluation before quarantine; DQ, evaluation during quarantine; POSTQ, evaluation after quarantine; CGA, comprehensive geriatric assessment; MMSE, Mini Mental State Examination.

This study is part of the GeroCovid protocol, a multi-purpose, multi-setting and multicenter initiative (8). GeroCovid involves individuals aged ≥ 60 years, prospectively or retrospectively observed since March 1st, 2020. Data are collected in multiple investigational sites in Italy and Norway, and recorded in a de-identified clinical e-Registry. This study (GeroCovid “GeroCovid dementia—psychological health cohort”) involves 10 Italian Centers for Cognitive Decline and Dementia (CDCDs) and considers three phases: before (PREQ, January–February), during (DQ, March-May) and after (POSTQ, July–December) quarantine. As for PREQ and POSTQ data were collected retrospectively and during the follow-up visits, respectively (see Figure 1 for schematic representation of the study). All the records were thus collected from January to December 2020. The study was approved by the BIO-CAMPUS Ethic Committee, University of Rome—Prot. Number: 22.5(20).20 OSS ComEt-UCBM. Each center, moreover, had the approval of its own Ethic Committee.

### Participants

Individuals with MCI or dementia were recruited from Italian CDCDs, according the following inclusion criteria: (1) last routine cognitive evaluation between January and March 2020 and next follow-up visit expected into 6–9 months; (2) Diagnosis of Alzheimer's Disease (AD) or other dementia, or diagnosis of MCI. Exclusion criteria were the following: (1) inability to undergo psychometrics tests for any reason; or, (2) history of psychiatric illness, according to clinical anamnesis. An initial number of 496 individuals meeting these criteria were originally contacted; of them, 260 agreed to participate to the study, although 10 did not complete the questionnaires. The total sample is thus composed of 250 participants.

### Procedure

For the purpose of this study, we considered participants' sociodemographic data (age, sex, years of education, cohabiting status), information on risk behaviors (smoking and alcohol consumption, Yes/No), medical history (including diagnosis of MCI and dementia, depressive mood and other coexisting chronic diseases), and drug treatments. The following comorbidities were considered: cardiovascular diseases (ischemic heart diseases, heart failure), atrial fibrillation, hypertension, cerebrovascular diseases, diabetes mellitus, dyslipidemia, thyroid dysfunctions, gastrointestinal diseases, cancer, osteoporosis, osteoarthritis, rheumatologic diseases, chronic obstructive pulmonary disease, hematologic disorders, chronic kidney disease, Parkinson's disease, vision deficits, hearing deficits. The total number of chronic diseases, calculated as the sum of the above-mentioned conditions, was used as comorbidity indicators.

A comprehensive geriatric assessment was performed in the PREQ and POSTQ phases. For this preliminary study, among the PREQ evaluations we considered cognitive performance through the Mini-Mental State Examination [MMSE (9)], nutritional status through the Mini-Nutritional Assessment [MNA (10)], and functional status through the Activities of Daily Living [ADL (11)] and Instrumental ADL scales [IADL (12)]. Concerning participants' social environment, we considered the presence of a formal or informal caregiver, and the number of informal visits received on average by each participant before the quarantine period.

DQ evaluations were carried-out by means of telephonic interviews to the patients, and included:

- Depression Anxiety Stress Scales-21 [DASS-21 (13)] composed by a set of three self-report scales designed to measure the emotional states of depression, anxiety and stress. Each of the three DASS-21 scales contains 7 items, divided into subscales with similar content. Cut-off scores for depression, anxiety, and stress were 10, 8, and 15, respectively (14). The depression scale (including the items 3, 5, 10, 13, 16, 17, and 21) assesses dysphoria, hopelessness, devaluation of life, self-deprecation, lack of interest/involvement, anhedonia and inertia. The anxiety scale (including the items 2, 4, 7, 9, 15, 19, and 20) assesses autonomic arousal, skeletal muscle effects, situational anxiety, and subjective experience of anxious affect. The stress scale is sensitive to levels of chronic non-specific arousal and included the items 1, 6, 8, 11, 12, 14, and 18). It assesses difficulty relaxing, nervous arousal, and being easily upset/agitated, irritable/over-reactive and impatient. Scores for depression, anxiety and stress are calculated by summing the scores for the relevant items, then multiplied by two. The cut-offs used to detect the presence of symptoms of depression
- Perceived Stress Scale [PSS-10; (15)] the most frequently used psychological measure to assess perceptions of stress. The degree to which the situations in a person's life are rated as stressful are evaluated by 10 items constructed to capture the level at which respondents perceive their lives as unpredictable, uncontrollable, or overloaded. The scale also contains a series of direct questions about current levels of perceived stress. The PSS was designed to be used in samples drawn from the general population with an educational level at least equal to lower middle school. The items and the response alternatives are easy to understand: for each item, respondents are asked to indicate how often they felt a certain way in the last month (“0 = Never,” “4 = Very often”). The PSS scores are obtained by reverse-scoring the responses to the four positively formulated items (items 4, 5, 7, and 8), then adding together the scores for each and every item. A short 4-item scale can be obtained using questions 2, 4, 5, and 10 of the 10 items in the PSS scale.
- Coping Orientation to Problems Experienced [COPE (16)] a multidimensional coping inventory to assess the different ways in which people respond to stress. Five scales (of four items each) measure conceptually distinct aspects of problem-focused coping (active coping, planning, suppression of competing activities, restraint coping, seeking of instrumental social support); five scales measure aspects of what might be viewed as emotion-focused coping (seeking of emotional social support, positive reinterpretation, acceptance, denial, turning to religion); and three scales measuring coping responses that arguably are less useful (focus on and venting of emotions, behavioral disengagement, mental disengagement).

The subscales are calculated as follows: the subscales “social support” (indicated by the sum of items 1, 10, 15, 18, 25), “positive attitude” (indicated by the sum of items 2, 6, 12, 16, 23, 24), “orientation to problem” (indicated by the sum of items 3, 5, 9,13, 20), and “transcendent orientation” (indicated by the sum of items 8, 11, 14, 19).

Finally, telephonic interviews were also performed to patients' caregivers to evaluate their burden through the Caregiver Burden Inventory [CBI; (17)] a 24-item self-report questionnaire for assessing the burden of caregivers caring for people with chronic disease. The items are rated on a 5-point Likert scale from 0 “Never” to 4 “Nearly always.” The questions cover 5 dimensions of caregiver burden: objective burden; time-dependence, referring to time demands for assistance; psychological burden, understood as the caregiver's feelings of exclusion from expectations and opportunities; physical burden, which describes the caregiver's feelings of fatigue and health problems; social burden, which describes the caregiver's feelings of role conflict; and emotional burden, which describes the caregiver's feelings of shame or embarrassment caused by the patient. Time spent for assistance, social involvement, physical involvement, and relational involvement are represented, respectively by the sum of the items from 1 to 5, from 6 to 10, 11 to 14, and 15 to 19.

The presence of a caregiver or appointed legal guardian (e.g., a support administrator) was always required during telephone interviews in order to limit potential biases due to patients' cognitive impairment and their ability to answer questions (18). All participants (or their caregivers or guardians) gave informed consent to their involvement to the study.

### Statistical Analyses

Descriptive characteristics of the sample are expressed as means ± standard deviations or as count (%), as appropriate. Participants were categorized according to their PREQ MMSE value (≥25, 23–24, and ≤ 22), and the comparison of the sociodemographic and health-related characteristics between such PREQ MMSE groups was performed through the ANOVA.

In order to test the hypothesis that individuals with worse cognitive performance (and their caregivers) may have been more strongly impacted by the COVID-19 quarantine, we first compared the DASS, PSS, COPE, and CBI scores (as continuous variables) between the three PREQ MMSE groups. To take into account the effect of potential confounders (i.e. age, sex, education, social environment, depressive mood, use of antipsychotics, and number of chronic diseases) in the association between PREQ MMSE with psychological well-being (depression, anxiety, stress), coping strategies, and caregiver burden, we run multivariable linear regressions. As independent variable, we considered PREQ MMSE either as continuous or categorical variable, in order to evaluate possible dose-response relationships. As dependent variables, we considered total DASS and PSS scores, the subscales “social support” (indicated by the sum of items 1, 10, 15, 18, 25), “positive attitude” (indicated by the sum of items 2, 6, 12, 16, 23, 24), “orientation to problem” (indicated by the sum of items 3, 5, 9,13, 20), and “transcendent orientation” (indicated by the sum of items 8, 11, 14, 19) of COPE, and the total CBI score and its subscales (time spent for assistance—sum of items 1 to 5, social involvement—sum of items 6 to 10, physical involvement—sum of items 11 to 14, relational involvement—sum of items 15 to 20) all as continuous variables.

## Results

Our sample included 250 individuals (62% women) with a mean age of 79.6 ± 6.7 years and a PREQ MMSE of 23.1 ± 2.8. The most frequent cognitive disorders in our sample were MCI (23.2%), mild AD (30.8%), and mild vascular dementia (21.6%). Comparing the characteristics of participants by cognitive performance (Table 1), we found that those with lower pre-quarantine MMSE were more likely to be older, women, to have lower educational level and functional status, with the need for a caregiver.

**Table 1:** Sociodemographic and health-related characteristics of participants by pre-quarantine cognitive functioning.

	**All (*n* = 250)**	**Pre-quarantine MMSE**			
**Characteristics**		**≥25 (*n* = 78)**	**23–24 (*n* = 72)**	**≤22 (*n* = 100)**	***p*-value**
Age (years)	79.6 ± 6.7	77.9 ± 6.6	79.6 ± 5.2	81.0 ± 7.4	0.007
Sex (female)	155 (62.0)	36 (46.2)	49 (68.1)	70 (70.0)	0.002
Years of schooling*					0.052
≤ 5	131 (52.4)	32 (41.0)	37 (51.4)	62 (62.0)	
6–8	66 (26.4)	23 (29.5)	19 (26.4)	24 (24.0)	
9–13	38 (15.2)	17 (21.8)	11 (15.3)	10 (10.0)	
>13	10 (4.0)	6 (7.7)	2 (2.8)	2 (2.0)	
Marital status*					0.008
Widowed	98 (39.4)	19 (24.4)	30 (42.3)	49 (49.0)	
Separated/divorced	9 (3.6)	1 (1.3)	1 (5.6)	4 (4.0)	
Single	10 (4.0)	2 (2.6)	4 (5.6)	4 (4.0)	
Partnered	56 (71.8)	33 (46.5)	43 (43.0)	132 (53.0)	
Social environment*					0.47
Living alone with <2 informal visits/w	11 (4.6)	4 (5.2)	3 (4.5)	4 (4.2)	
Living alone with ≥2 informal visits/w	39 (16.3)	7 (9.1)	15 (22.4)	17 (17.7)	
Not living alone	188 (78.3)	65 (84.4)	49 (73.1)	74 (77.1)	
Living in nursing home	2 (0.8)	1 (1.3)	0 (0.0)	1 (1.0)	
Caregiver*					<0.001
No	35 (14.5)	23 (29.9)	9 (13.4)	3 (3.1)	
Informal	201 (83.4)	54 (70.1)	56 (83.6)	91 (93.8)	
Formal	5 (2.1)	0 (0.0)	2 (3.0)	3 (3.1)	
Living with caregiver	128 (51.2)	35 (44.9)	36 (50.0)	57 (57.0)	0.04
Smoking habits*					0.38
Never	191 (79.7)	56 (71.8)	53 (74.6)	82 (82.0)	
Former	42 (16.9)	7 (9.0)	6 (8.5)	3 (3.0)	
Current	16 (6.4)	15 (19.2)	12 (16.9)	15 (15.0)	
Alcohol consumption*					0.31
Abstemious	191 (77.0)	55 (70.5)	53 (75.7)	83 (83.0)	
Light-to-moderate	55 (22.2)	22 (28.2)	16 (22.9)	17 (17.0)	
Heavy	2 (0.8)	1 (1.3)	1 (1.4)	0 (0.0)	
Phisical activity ≥4 h/w	45 (18.0)	16 (20.5)	13 (18.1)	16 (16.0)	0.25
Cognitive disorder*					0.01
MCI	58 (23.2)	30 (38.5)	15 (20.8)	13 (13.0)	
AD	77 (30.8)	16 (20.5)	24 (33.3)	37 (37.0)	
VD	54 (21.6)	18 (23.1)	14 (19.4)	22 (22.0)	
Other	44 (17.6)	12 (15.4)	13 (18.1)	19 (19.0)	
Use of antipsychotics	50 (20.0)	9 (11.5)	13 (18.1)	28 (28.0)	0.04
Depressive mood	43 (17.2)	15 (19.2)	11 (15.3)	17 (17.0)	0.48
Hearing deficits	24 (9.6)	8 (10.3)	5 (6.9)	11 (11.0)	0.65
Vision deficits	26 (10.4)	6 (7.7)	6 (8.3)	14 (14.0)	0.31
ADL	4.7 ± 1.4	4.9 ± 1.3	5.0 ± 1.1	4.2 ± 1.4	<0.001
IADL (men)	2.8 ± 1.6	3.4 ± 1.7	2.6 ± 1.2	2.1 ± 1.3	0.002
IADL (women)	4.1 ± 2.5	5.6 ± 2.6	4.5 ± 1.9	3.1 ± 2.4	<0.001
MNA	11.2 ± 2.5	11.6 ± 2.3	11.3 ± 2.2	10.8 ± 2.7	0.12
N. chronic diseases	2.7 ± 1.8	2.8 ± 1.9	2.3 ± 1.7	2.8 ± 1.8	0.23

Numbers are mean ± SD, or count (%), as appropriate. MCI, mild cognitive impairment; AD, Alzheimer's disease; VD, vascular dementia; ADL, activities of daily living; IADL, instrumental ADL; MNA, Mini-Nutritional Assessment; w, week. *Frequencies do not sum to 100% due to missing values.

Symptoms of anxiety, depression, and psychological stress (DASS) were observed in 28.8, 48, and 24.8% of the sample, respectively (for cut-off scores see the Methods section). As shown in Table 1, individuals cognitively more impaired (MMSE ≤ 22) showed higher total DASS and PSS scores, compared both to individuals with MMSE ≥25 and 22 < MMSE <25. As for the COPE scale, individuals with greater cognitive impairment showed worse “positive attitude” and “problem orientation” coping strategies, as compared to those with higher MMSE scores. As expected, higher caregiving burden, in particular as for time spent for assistance (items 1–5) was observed in caregivers of individuals with more severe cognitive impairment (Table 2).

**Table 2:** Psychological well-being and coping strategies scales in the total sample and by pre-quarantine cognitive functioning.

		**DASS total score**	**PSS total score**	**COPE ∑3 positive attitude**	**COPE ∑4 orientation to problem**	**CBI total score**	**CBI ∑ 1–5**
All (*n* = 250)		14.4 ± 11.4	16.2 ± 6.9	15.4 ± 4.3	12.1 ± 3.4	21.1 ± 15.9	7.6 ± 5.4
MMSE	≥25 (*n* = 78)	11.3 ± 9.4	13.8 ± 6.8	16.5 ± 3.6	13.2 ± 3.1	17.1 ± 14.6	5.9 ± 5.5
	23-24 (*n* = 72)	15.5 ± 11.8	16.9 ± 6.6	15.6 ± 4.1	12.0 ± 3.1	19.5 ± 14.7	6.8 ± 5.1
	≤ 22 (*n* = 100)	16.1 ± 12.3	17.5 ± 6.8	14.5 ± 4.8	11.3 ± 3.7	24.1 ± 16.6	9.0 ± 5.3
*p*-value		0.02	0.001	0.01	0.002	0.04	0.002

*Numbers are mean values ± standard deviation. P-values refer to the comparisons between MMSE groups. DASS, Depression Anxiety Stress Scales; PSS, Perceived Stress Scale; COPE, coping strategies inventory; ∑3 = sum of the items 2, 6, 12, 16, 23, 24 indicating positive attitude; ∑4 = sum of the items 3, 5, 9, 13, 20 indicating orientation to problem; CBI, caregiver burden inventory*.

The linear regression models confirmed that lower cognitive functioning was independently associated with a stronger negative psychological and affective reaction to quarantine, as well as with a poorer implementation of coping strategies, and with higher caregiving burden (Table 3). Specifically, higher stress was reported by caregivers in association with a reduction of the time dedicated to themselves, a greater sense of failure of hopes and expectations, and physical involvement (Items 1–5, 6–10, and 11–14 of CBI, respectively). No substantial differences were observed when testing the association between PREQ MMSE and other COPE and CBI subscales (data not shown).

**Table 3:** Linear regression models on the association between pre-quarantine MMSE and patients' psychological well-being and caregivers' burden during quarantine.

	**β** **coefficient (95% confidence interval), p-value**							
**PREQ MMSE**	**DASS total score**	**PSS total score**	**COPE ∑3**	**COPE ∑4**	**CBI ∑ 1–5**	**CBI ∑ 6–10**	**CBI ∑ 11-14**	**CBI total score**
**Total score**								
Per each 1-point increase	−0.7 (−1.3; −0.04) *p =* 0.04	−0.7 (−1.1; −0.4) *p < * 0.001	0.3 (0.1; 0.6) *p =* 0.006	0.3 (0.2; 0.5) *p < * 0.001	−0.7 (−1.0; −0.4) *p < * 0.001	−0.5 (−0.8; −0.2) *p =* 0.001	−0.3 (−0.5; −0.1) *p =* 0.01	−1.7 (−2.6; −0.8) *p < * 0.001
**Categorical variable**								
≥25	[ref]	[ref]	[ref]	[ref]	[ref]	[ref]	[ref]	[ref]
23–24	4.2 (0.3; 8.1) *p =* 0.04	3.2 (0.9; 5.5) *p =* 0.007	−0.6 (−2.0; 0.9) *p =* 0.42	−0.9 (−2.1; −0.2) *p =* 0.12	1.2 (−0.8; 3.2) *p =* 0.24	1.4 (−0.6; 3.4) *p =* 0.17	0.5 (−1.0; 1.9) *p =* 0.55	3.3 (−3.3; 10.0) *p =* 0–32
<23	4.4 (0.6; 8.2) *p =* 0.02	4.1 (1.9; 6.3) *p < * 0.001	−1.8 (−3.2; −0.5) *p =* 0.009	−1.7 (−2.8; −0.6) *p =* 0.002	3.2 (1.3; 5.1) *p =* 0.001	2.3 (0.4; 4.2) *p =* 0.02	0.9 (−0.5; 2.3) *p =* 0.22	6.7 (0.8; 12.7) *p =* 0.03

*Models are adjusted for age, sex, education, social environment, depression, use of antipsychotics, number of chronic diseases. DASS, Depression Anxiety Stress Scales; PSS, Perceived Stress Scale; COPE, coping strategies inventory; ∑3 = sum of the items 2, 6, 12, 16, 23, 24 indicating positive attitude; ∑4 = sum of the items 3, 5, 9, 13, 20 indicating orientation to problem. CBI, Caregiver Burden Inventory. ∑ [1–5] = sum of the items from 1 to 5 indicating time spent for assistance; ∑ [6–10] sum of the items from 6 to 10 indicating social involvement ∑ [11–14] sum of the items from 11 to 14 indicating physical involvement; ∑ [15–19] sum of the items from 15 to 19 indicating relational involvement; PREQ MMSE, pre-quarantine Mini-Mental State Examination*.

## Discussion

This study showed that, in individuals with MCI and dementia, the more severe the cognitive impairment, the higher the depression and anxiety experienced during the first wave of quarantine due to COVID-19. This evidence suggests that, despite the potential lack of awareness on the pandemic, individuals with dementia did perceive distress during the quarantine period. In particular, they showed higher scores in those items investigating psycho-somatic symptoms (“I feel my mouth dry” and “I feel nervous”). These symptoms are reported to represent psychological distress expressed through physical disturbances by individuals unable to express their emotions due to genetic and environmental factors (19). What is more, this finding is also corroborated by an increasing and challenging literature attesting that somatic disorders in individuals with dementia are related to outcomes and quality of life (20). Interestingly, among the most stressful events in life (i.e., spouse or relative death illness/surgical interventions, or problems with the family), people with dementia also reported as strongly demanding and tense “change in environment” (21), which exactly is what happen with Covid-19 occurrence. Similarly, Giebel et al. (22) found that social care and support services changes and closures altered the typical physical and “communal” environment negatively impacting on psychological well-being of people with dementia.

Our results are, therefore, in line with other studies: as recently reviewed by Sepulveda-Loyola et al. (23) in their meta-analysis, several consequences on mental health occur along with pandemics, such as depression, emotional disturbances, stress, deflection of mood, irritability and insomnia. Alarmingly, Yip et al. (24) also showed that these disorders are associated with higher suicide rates during pandemics, particularly in older adult populations.

In our study, individuals with more severe cognitive impairment were also found to have poorer coping strategies than those with higher cognitive performance. One can argue that this finding is not strictly linked to the pandemic or to the current cognitive status of participants (25), and unfortunately, we had not detailed information on psycho-affective distress level before quarantine. Yet the association remained significant even after adjusting for the presence of PREQ depression. Therefore, it is possible that poorer and less efficient coping strategies exposed the individuals to higher social distancing-associated distress (26). On the whole, subjects with more severe cognitive impairment, in spite of a limited awareness of pandemic-related issues, are not protected from the deleterious psychological effects of COVID-19, as also confirmed by Boutoleau-Bretonnière et al. (27). It is well-known that older adults are at increased risk of being socially isolated compared to younger adults under normal conditions (28). Similarly, people living with cognitive impairment are particularly subjected to the effects of social isolation that negatively impact their cognitive and affective well-being (29). The pandemic seems thus to have done nothing but worsen an already existing framework of frailty typical of this population (30, 31). Interestingly, in our study this happens in the most cognitively impaired individuals that might be considered as already so compromised that social isolation cannot hit them furtherly. Instead, social and environmental stimuli appear to be still important even in the more advanced stages of dementia. This result stresses the importance of supporting individuals with dementia through cognitive stimulation trainings and not just with pharmacological treatments (32).

As expected, these consequences did not seem to affect only patients, but also their caregivers. Indeed, also in the context of COVID-19 quarantine, we found that the greater the cognitive impairment of the patients, the heavier the burden of their caregivers. Our results confirm the other few studies available that suggest an increase of global caregiver burden during the COVID-19 (33–35). Our study, moreover, adds some novelty to the current literature by highlighting that the main burden experienced is related to psychological affliction, more than physical or time-dependent assistance. Our results only partially are in accordance with those reported by Cohen et al. **(author?)** (36), according to whom family members' main concern was, for severe dementia cases, fear of absence of the paid caregiver during the pandemic. For mild cases, instead, caregivers mainly reported fear of spreading the disease while assisting their relatives with instrumental activities.

### Limitations and Strength

Some limitations should be acknowledged. First of all, the scales used for the psycho-affective evaluation are not validated for the remote administration. However, although originally not designed in this format, these tools have been previously administered remotely (37). Secondary, the psycho-affective profile of patients with cognitive deterioration before the pandemic is not known, we only have reference population data. However, as stated in the Methods section, this is a preliminary study and a more complete, longitudinal one (Gerocovid initiative) will show, at least, cognitive change (measured by MMSE) before and after the pandemic. Similarly, pre-pandemic anamnestic and clinical data were collected retrospectively. Finally, our results should be considered as limited to mild and moderate dementia and not generalizable to individuals with more severe cognitive impairment. Conversely, the research topic is timely and brings novelty to the COVID-19 literature. Different aspects of psychological well-being are considered, including the precious, though sometimes neglected, caregivers' point of view.

## Conclusions

This study found that during the Covid-19 pandemic, people with neurocognitive impairments seem to experience, psycho-affective disorders, which vary according to their pre-quarantine cognitive functioning. In particular, patients with more severe cognitive impairment psychologically seem to have suffered the most of the effects of the pandemic, as well as their caregivers. Our study points out the role of poor and dysfunctional coping strategies adopted by individuals with MCI and dementia to explain the distress related to Covid-19 pandemic. Limited positive attitude and orientation to problem behaviors in facing the pandemic further contribute to the stress response. In conclusions, physicians and health care professionals caring for people with neurocognitive disorders should be aware that cognitive impairment does not prevent from the negative effects of the pandemic on emotional and affective distress. Attention should be given to the psychological well-being of individuals with MCI or dementia, and of their caregivers.

## Data Availability Statement

The raw data supporting the conclusions of this article will be made available by the authors, without undue reservation.

## Ethics Statement

The studies involving human participants were reviewed and approved by BIO-CAMPUS Ethic Committee, University of Rome – Prot. Number: 22.5(20).20 OSS Com-Et-UCBM. The patients/participants provided their written informed consent to participate in this study.

## Author Contributions

All authors listed have made a substantial, direct and intellectual contribution to the work, and approved it for publication.

## Conflict of Interest

SS is shareholder and GZ is shareholder and employee of Bluecompanion Ltd (London, UK). The remaining authors declare that the research was conducted in the absence of any commercial or financial relationships that could be construed as a potential conflict of interest.

## Publisher's Note

All claims expressed in this article are solely those of the authors and do not necessarily represent those of their affiliated organizations, or those of the publisher, the editors and the reviewers. Any product that may be evaluated in this article, or claim that may be made by its manufacturer, is not guaranteed or endorsed by the publisher.
